# Metal Complexation of Arabinoxylan Engenders a Smart Material Offering pH, Solvents, and Salt Responsive On–Off Swelling with the Potential for Sustained Drug Delivery

**DOI:** 10.3390/gels8050283

**Published:** 2022-05-02

**Authors:** Syed Nasir Abbas Bukhari, Muhammad Ajaz Hussain, Muhammad Tahir Haseeb, Abdul Wahid, Naveed Ahmad, Syed Zajif Hussain, Rizwan Nasir Paracha, Muhammad Usman Munir, Mervat A. Elsherif

**Affiliations:** 1 Department of Pharmaceutical Chemistry, College of Pharmacy, Jouf University, Al Jouf, Sakaka 72388, Saudi Arabia; mumunir@ju.edu.sa; 2 Institute of Chemistry, University of Sargodha, Sargodha 40100, Pakistan; abdulchemist55@gmail.com; 3 College of Pharmacy, University of Sargodha, Sargodha 40100, Pakistan; mtahir212@yahoo.com; 4 Department of Pharmaceutics, College of Pharmacy, Jouf University, Al Jouf, Sakaka 72388, Saudi Arabia; nakahmad@ju.edu.sa; 5 Department of Chemistry and Chemical Engineering, Lahore University of Management Sciences, Lahore 54792, Pakistan; syed.hussain@lums.edu.pk; 6 Department of Chemistry, Thal University Bhakkar, Bhakkar 30000, Pakistan; rizwanparacha@gmail.com; 7 Chemistry Department, College of Science, Jouf University, Al Jouf, Sakaka 72388, Saudi Arabia; maelsherif@ju.edu.sa

**Keywords:** crosslinked arabinoxylan, polysaccharide, pH responsive, stimuli responsive, swelling–deswelling, sustained release

## Abstract

The present study aimed to develop a stable interconnected matrix as a sustained release drug delivery material. Arabinoxylan (AX) was extracted from ispaghula husk and then crosslinked with different concentrations, i.e., 0.5, 1.0, and 1.5 g of CaCl_2_ per 0.25 g of AX. The crosslinking was confirmed through Fourier transform infrared spectroscopy. The swelling capacity of crosslinked AX (CL-AX) was evaluated against buffer solutions of pH 1.2, 6.8, 7.4, and water. The swelling capacity increased from pH 1.2 to pH 7.4 and followed the second order swelling kinetics. The swelling study also revealed that CL-AX with 1.0 g CaCl_2_ showed maximum swelling capacity. The swelling–deswelling (on–off switching) behavior of CL-AX was evaluated in water–ethanol, water–0.9% NaCl solution, and buffer solutions of pH 7.4–1.2 and showed responsive swelling–deswelling behavior. Scanning electron microscopy revealed a highly porous nature of CL-AX with a mesh of thin fibrous networking. Hemocompatibility studies of CL-AX revealed its non-thrombogenic and nonhemolytic attributes. The CL-AX matrix tablet prolonged the release of enalapril maleate for 24 h, and the drug release followed the zero order kinetics and super case-II transport mechanism. Therefore, CL-AX can be recognized as a stimuli responsive and hemocompatible biomaterial with sustained drug release potential.

## 1. Introduction

**Figure 1:**
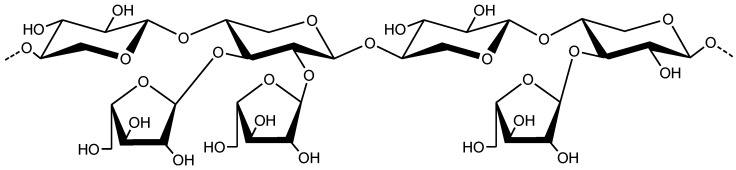
Structure of arabinoxylan.

*Plantago ovata* seed husk or psyllium (Syn: Isapgol, Ispaghula, Sand Plantain and Spogel) is composed of fiber commonly used to cure gastric disorders, i.e., diarrhea, constipation, and ulcerative disorders [1,2,3]. Upon soaking in water, psyllium extrudes mucilage, and the main component of this mucilage has been recognized as arabinoxylan (AX) [4]. The AX has a branched structure in which the linear chains consist of β-1,4-linked D-xylopyranose and branches of α-L-arabinofuranose (Figure 1). Moreover, the prebiotic potential of psyllium has been established in clinical trials; therefore, AX exhibits many pharmaceutical, medicinal, and biomedical applications [5]. 

Crosslinking of the polymeric material with metal ions can impart required properties in the polymeric network [6]. The intensity of these properties mainly depends upon the conditions of the reaction medium, i.e., temperature, pH, ionic strength, etc. Many polysaccharides and their derivatives are now being crosslinked using polyvalent cations, i.e., Ca^2+^, Mg^2+^, Ti^3+^, Cu^2+^, and Al^3+^. Alginate, pectin, curdlan and chitosan are crosslinked with calcium ions to develop different pharmaceuticals and healthcare materials [7]. The crosslinking of these polysaccharides results in the formation of scaffolds, multifold gels, and highly porous materials that could be a material of choice for the development of targeted/sustained drug release [8,9,10].

Herein, we prepare a crosslinked polysaccharide (arabinoxylan) gel using Ca^2+^ through an interfacial complexation technique. The new material, crosslinked arabinoxylan (CL-AX), will be evaluated for stimuli-responsive dynamic swelling and/or swelling–deswelling (on–off switching) properties. As previous work showed that AX upon swelling shows interconnected channels, the aim is to observe the surface morphology and a deep look into the polymeric network through scanning electron microscopy (SEM) after crosslinking [11]. The newly developed material will be appraised for sustained/targeted drug release studies at the optimum pH of the gastrointestinal tract, especially at intestinal pH 6.8. We focus on performing some preliminary experiments to evaluate the biocompatibility of CL-AX.

## 2. Results and Discussion

### 2.1. Isolation of AX

AX was isolated using a hot water extraction method, which is considered an efficient method for high yield of swellable polysaccharides. The percentage yield of isolated AX from ispaghula husk was 14.9 wt%. The AX powder obtained after drying was light brown and fluffy.

### 2.2. Crosslinking of AX

The CL-AX was formed when a certain amount of CaCl_2_ was allowed to mix with AX. The reaction conditions and results are summarized in Table 1. CaCl_2_ was mixed with AX at room temperature and refluxed under stirring. All the concentrations (5, 10, 15% CaCl_2_ vs. 0.25 g of AX powder) resulted in gel formation by the interfacial complexation of Ca^2+^ ions. The swelling of CL-AX in deionized water (DW) was improved by increasing the concentration of CaCl_2_ from 0.5 to 1.0 g per 0.25 g of AX, while it decreased for 1.5 g per 0.25 g of AX. This was due to the extent of the entanglement and the retractive force within the polysaccharide, which increases when the concentration of crosslinking agent increases; as a result, swelling decreases [12]. Due to these two factors, gel did not let the water penetrate in; hence, limited swelling resulted.

### 2.3. Characterization 

#### 2.3.1. FTIR Spectroscopy

**Figure 2:**
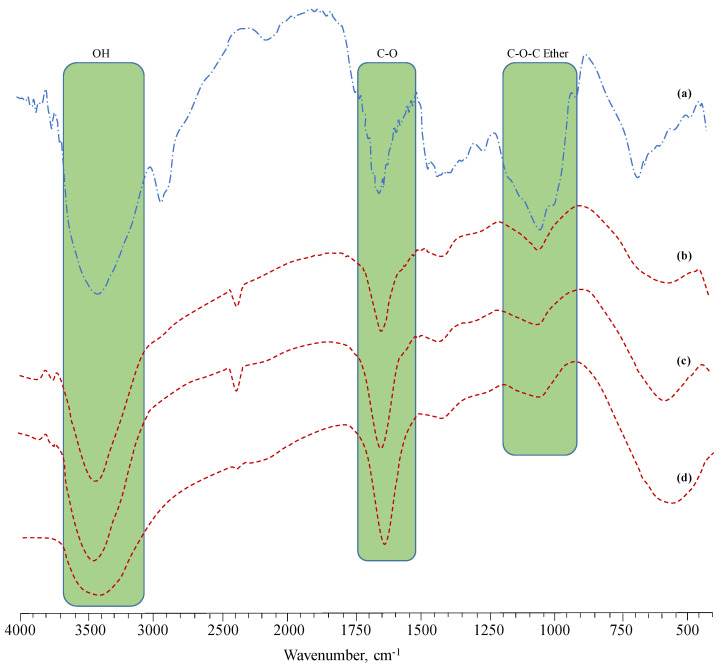
FTIR (KBr) spectra of AX (**a**), CL-AX1 (**b**), CL-AX2 (**c**), and CL-AX3 (**d**).

FTIR spectra of AX and CL-AX were recorded and absorption bands were assigned by comparing it with the FTIR of AX available in the literature [13]. The bands observed in the FTIR spectra were assigned as: 3460–3350 cm^−1^ in AX and 3429 cm^−1^ in CL-AX (OH stretching as broad band), 2926 cm^−1^ (aliphatic saturated CH stretching) (δ CH_2_), 1408 cm^−1^ CH_2_ scissors vibration, 1290–1253 cm^−1^ δ_Asym_ bridged oxygen, 1064–1045 cm^−1^ (υ C-C, υ C-O), and 669–555 cm^−1^ polymer back bone. The band appearing around 2400 cm^−1^ corresponded to the absorbing moisture. The C-O band of AX (1643 cm^−1^) after the crosslinking with CaCl_2_ appeared as a strong signal around 1636–1637 cm^−1^. Moreover, the gradual decrease and increase in the band height of the OH stretching (3400–3600 cm^−1^) and carbonyl (CO) asymmetric stretching bands in the FTIR spectra of CL-AX1 to CL-AX3 also confirmed the successful crosslinking of the AX. The FTIR spectra of the AX powder, CL-AX1, CL-AX2, and CL-AX3 are shown in Figure 2.

#### 2.3.2. SEM

**Figure 3:**
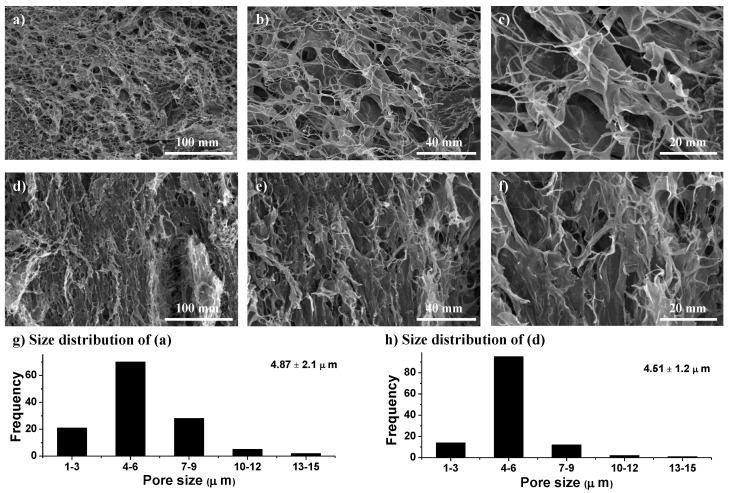
Scanning electron micrographs of transverse (**a**–**c**) and longitudinal (**d**–**f**) cross sections of swollen then freeze-dried CL-AX2 (with average pore size 4.51 ± 1.6 µm) at different magnifications. Size distribution of macropores (**g**,**h**); transverse (**a**) and longitudinal (**d**) cross sections.

SEM images of the swollen and then freeze-dried CL-AX are depicted in Figure 3. The images show well-distributed, organized, and interconnected macrochannels. These pores and interconnected channels absorbed a significant amount of water, which resulted in the swelling of the CL-AX. As compared to the SEM images of AX [11], CL-AX had evenly distributed and well interconnected channels/macropores. Moreover, the pore size was reduced to 4.51 µm in CL-AX from 6 µm as observed in AX. Therefore, the structured distribution of macropores in CL-AX was likely due to the crosslinking of the polymeric chains. 

### 2.4. pH Responsive Swelling of CL-AX

**Figure 4:**
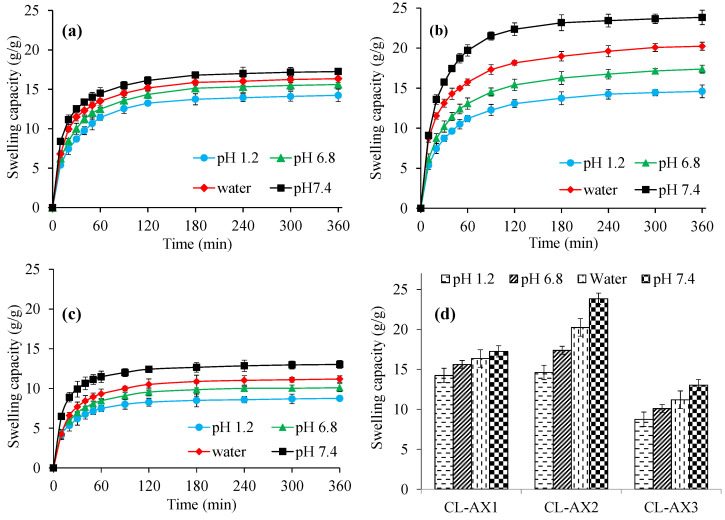
Swelling capacity of CL-AX1 (**a**), CL-AX2 (**b**), and CL-AX3 (**c**) in deionized water and different pH along with viability graphs (**d**) (*n* = 6).

Hydrogels respond differently when exposed to various physical factors such as ionic strength, electric field, pH, and temperature and have exhibited swelling/deswelling and immediate/sustained drug release [14,15,16,17]. Therefore, the swelling studies of CL-AX were carried out in buffers of pH 1.2, 6.8, 7.4, and in deionized water simulating the pH of stomach, small intestine, and large intestine, respectively (Figure 4). More swelling of CL-AX was observed in the buffers of pH 6.8, 7.4, and deionized water rather than the acidic buffer (pH 1.2). In buffers of pH 6.8 and 7.4, ionization of carboxylic groups occurred which resulted in anion–anion repulsion, hence, the distance increased between the polymeric chains resulting in high swelling of CL-AX. Moreover, CL-AX in the buffer of pH 7.4 showed more swelling than the deionized water. At slightly basic pH 7.4, the COOH groups became ionized, and due to the increased anion–anion repulsion, the swelling of CL-AX increased as compared with water. Whereas, the charge screening effect of the excessive cations produced at pH 6.8 resulted in the shielding of the carboxylate anions, hence, reducing the anion–anion repulsion, causing relatively less swelling at pH 6.8 as compared with water [18,19]. It has been reported that AX contains some free carboxylic acid functional groups available on polymeric chains which offer charge screening with divalent calcium ions. In an acidic condition, protonation of the carboxylic group (present in the polymer chain) resulted in the significantly decreased repulsion among polymer chains. Therefore, the swelling capacity of the CL-AX was a bit reduced at pH 1.2. It was also noticed that the swelling capacity of CL-AX2 (Figure 4b) was high compared to CL-AX1 (Figure 4a) and CL-AX3 (Figure 4c). Therefore, CL-AX2 formulation was upsized and used for SEM analysis and other characterization techniques, i.e., swelling–deswelling, biocompatibility, and in vitro drug release studies. The viability graph of all three formulations indicated the swelling capacity after 6 h at different pH and in water (Figure 4d). Owing to the pH dependent high swelling capacity, CL-AX can be considered a potential candidate for many pharmaceutical applications, especially the development of sustained or targeted drug delivery formulations. 

### 2.5. Swelling Kinetics

**Figure 5:**
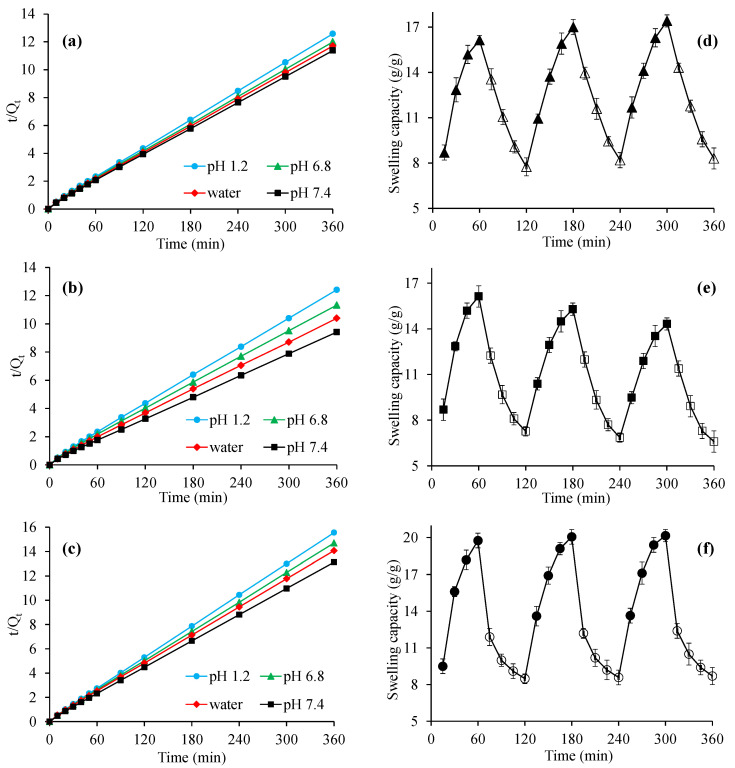
Second-order swelling kinetics of CL-AX1 (**a**), CL-AX2 (**b**), and CL-AX3 (**c**) and the swelling–deswelling behavior of CL-AX2: on–off switching in deionized water (DW) and ethanol (**d**), DW and 0.9% NaCl solution (**e**), and in a buffer solution of pH 7.4 and pH 1.2 (**f**), respectively (*n* = 3).

The diffusion of the solvent controls the swelling of a swellable material, and the swelling kinetic study of such materials is explained by a second order kinetic model. Swelling data obtained from the swelling capacity of CL-AX in water, buffers of pH 1.2, 6.8, and 7.4 were used to determine the swelling kinetics. The results of the kinetic studies showed that the swelling data closely resembled second order kinetics as indicated by the straight line (Figure 5). Second order swelling kinetics is usually used to explain the rate of swelling with respect to the swelling capacity of a polymeric material in swollen (hydrated) and non-swollen (dehydrated) conditions. Furthermore, second order swelling kinetics has also demonstrated that the rate of absorbency of a swelling media is directly proportional to the capacity of a polymer to convert into a hydrated (swollen) or dehydrated (non-swollen) form at a specific time [20,21]. Therefore, second order swelling kinetics is considered an appropriate way to explain the swelling capability and rate of absorbency of a polymeric material.

### 2.6. Swelling–Deswelling Response to External Stimuli

#### 2.6.1. Deionized Water and Ethanol

Swelling of CL-AX2 was observed when it was immersed in water, while CL-AX2 showed deswelling when it was soaked in ethanol (Figure 5d). The literature reported that the concurrent administration of alcohol and sustained release dosage forms containing a polymeric material as a matrix system adversely affects the release of drugs [22,23]. Hence, the therapeutic efficiency of the drugs can be compromised. Therefore, since one objective is to use the CL-AX2 as a sustained release material in tablets, the swelling capacity of this material was observed in water and ethanol. The reason for the deswelling of CL-AX2 in ethanol is associated with the low polarity and dielectric constant of ethanol and water, i.e., 24.55 and 80.40, respectively, which resulted in fewer hydrogen bonds with AX. The ionization of the ionizable groups is another important factor, which was decreased in ethanol and proportionally reduced the swelling. Moreover, the ionization was reduced due to the low dielectric constant; hence, it decreased the swelling capacity of the CL-AX2. The extensive hydrogen bonding capability of CL-AX2 with water and swift washing out of ethanol are considered the reasons for CL-AX2 swelling.

#### 2.6.2. Deionized Water and NaCl Solution

CL-AX2 was suspended in water and then in solution of NaCl (0.9%) for the study of swelling and deswelling, respectively. CL-AX2 showed swelling in water, and the reverse effect, i.e., deswelling was observed when it shifted to the NaCl solution (Figure 5e). In NaCl solution, the osmotic pressure increases, which results in the withdrawal of water from the swollen CL-AX2 and letting it shrink. Moreover, the deswelling of CL-AX2 in normal saline could also be attributed to the charge screening effect of additional cations present in the saline solution.

#### 2.6.3. Basic and Acidic Buffers

Swelling of CL-AX2 was observed in the basic buffer (pH 7.4), while deswelling was noticed in the acidic buffer (pH 1.2), and the results of these on–off experiments are shown in Figure 5f. The mechanism of the swelling–deswelling behavior of the CL-AX2 has already been discussed in a previous section. 

As compared to the previously reported swelling–deswelling studies of unmodified AX, the deswelling ability of the crosslinked formulation (CL-AX2) was increased which lead to more distinct on–off behavior. It was also observed that the reported formulation has the ability to completely deswell to its initial level. Whereas, in non-crosslinked material (AX), it was difficult to completely deswell to its initial level in case of water/normal saline and at pH 7.4/1.2 [11]; hence, the on–off switching behavior of CL-AX2 was better than its precursor AX, due to its crosslinking. Therefore, it is concluded that CL-AX2 is more stimuli-responsive than AX.

### 2.7. Biocompatibility Studies

The value of the hemolytic index was calculated at 2.71%. According to the safety standards of ISO document 10993–3 2002, a material having a hemolytic index <5% is recognized as safe and can be used for biomedical applications [24]. The thrombogenicity potential of CL-AX2 was evaluated by determining the weight of a blood clot compared to the control. The weight of the formed blood clot and the percentage of thrombose were calculated and found to be 0.39 ± 0.03 g and 93.79 ± 1.97%, respectively (Table 2). In positive control, the weight of the blood clot was more than the sample. These results of biocompatibility studies indicated the non-thrombogenic and nonhemolytic nature of CL-AX2. Hence, CL-AX2 can be used for the development of various biomedical products. 

### 2.8. In Vitro Drug Release, Kinetics, and Release Mechanism

**Figure 6:**
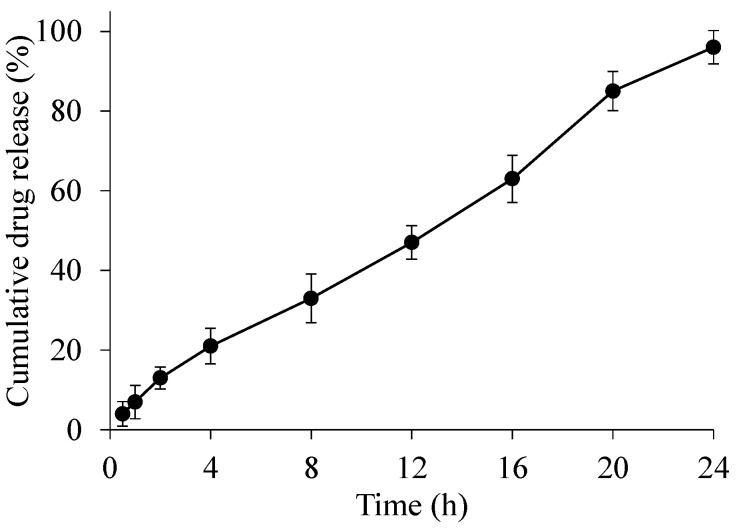
Drug release study from CL-AX2 based tablets at pH 6.8.

A drug release study was performed at pH 6.8, i.e., the pH of the small intestine, in order to evaluate the drug release behavior (Figure 6). During a 24 h study, a sustained drug release pattern was observed, and almost 85% of the drug was released in 20 h. A drug release study of enalapril maleate indicated that the CL-AX2 has the potential to be used as sustained release material in tablet formulations. Drug release kinetics applied on the data revealed that the drug release followed zero order kinetics (R^2^ = 0.9911), which indicated that the release was independent of the concentration of the drug in the polymeric matrix, i.e., the tablet (Table 3). As compared to a previously reported drug release study of an AX based tablet formulation [11], the R^2^ value of the zero order was 0.8664. Whereas, in case of CL-AX2, the R^2^ value was calculated as 0.9911. which was much better than the previous study. Hence, the CL-AX2 appeared to be a better material for the development of sustained release drug delivery system with possible zero order kinetics as compared to AX. Moreover, the drug release mechanism followed the super case-II transport mechanism (*n* = 0.923) as revealed by the Korsmeyer-Peppas model, which showed that the release was governed by the erosion of the polymeric matrix [25,26]. 

## 3. Conclusions

Crosslinking of AX resulted in an interlinked, channeled, and strong porous gel, which has the potential to be used in the pharmaceutical industry as a drug carrier. AX hydrogel showed high swelling in deionized water, at pH 6.8, and 7.4, while unable to show reasonable swelling at pH 1.2. Furthermore, the excellent stimuli-responsive swelling–shrinking behavior of the CL-AX hydrogel in water and ethanol, in basic (pH 7.4) and acidic (pH 1.2) media, and in water and normal saline solution has proven its potential as an intelligent drug delivery system. The swelling–deswelling behavior of the crosslinked hydrogel showed that it is a superabsorbent material, which can be used for potential pharmaceutical applications. The drug release from CL-AX revealed a sustained release pattern following the zero order kinetics, which made it an important excipient for a sustained release drug delivery system. More importantly, CL-AX proved to be hemocompatible material, which broadens its application in the biomedical field. 

## 4. Materials and Methods

### 4.1. Materials

Ispaghula husk was purchased from Marhaba Laboratories Pvt. Ltd., Lahore, Pakistan. Calcium chloride (CaCl_2_), ethanol, hydrochloric acid, and sulfuric acid were used as received from E. Merck, Germany. Sodium chloride, n-hexane, potassium dihydrogen phosphate, and potassium chloride were obtained from Riedel-de Haën, Germany. Sodium hydroxide of analytical grade was acquired from Merck and standardized with oxalic acid before further use. The enalapril maleate used in this research was according to the standard of the United States Pharmacopeia (USP). Deionized water (DW) was used all through the work. 

### 4.2. Isolation of Arabinoxylan

Arabinoxylan (AX) was isolated from ispaghula husk using an already reported method with slight modifications [4]. Briefly, ispaghula husk (500 g) was cleaned physically and kept overnight in distilled water maintaining a pH of 12 through NaOH solution (2.5% *w*/*v*). AX was isolated through vacuum filtration and then coagulated at pH 3 using concentrated acetic acid. After that, the AX was washed several times with *n*-hexane to remove lipophilic substances and then with DW until a constant pH was obtained. The AX was freeze-dried, ground to a fine powder, and stored in an air tight container until further use.

### 4.3. Crosslinking of AX

AX powder (0.25 g) was mixed in water (5 mL) and allowed to suspend before crosslinking. Different concentrations of CaCl_2_, i.e., 5, 10, and 15% *w*/*v*, were used for the crosslinking of AX. For each concentration of CaCl_2_, 5% *w*/*v* AX suspension (0.25 g in 5 mL water) was used.

In a typical reaction, 5 mL of AX suspension was mixed with 10 mL of CaCl_2_ solution (5% *w*/*v*). The reaction mixture was boiled and allowed to stand overnight for the completion of the crosslinking process, which resulted in a viscous/thick gel. The prepared gel was separated after filtration and dried at 50 °C for 48 h. The dried crosslinked AX gel (CL-AX) was stored in a desiccator in the presence of silica gel to avoid any possible water absorption. The same procedure was adopted for the synthesis of CL-AX using the remaining concentrations of CaCl_2_ (see Table 1). 

### 4.4. Characterization

#### 4.4.1. FTIR Spectroscopy

The synthesis of CL-AX was confirmed through FTIR and performed on an IR prestige-21 (Shimadzu, Japan) using the KBr pellet technique. The CL-AX was dried in a hot air oven at 40 °C for 72 h and then mixed with KBr. A thin disk of the sample was prepared using a hydraulic press and then dried in a hot air oven for 1 h prior to analysis. The FTIR spectra were recorded in the range of 4000–400 cm^−1^ with 20 scans and adjusting the resolution of 4 cm^−1^.

#### 4.4.2. Scanning Electron Microscopy

The surface morphology of the CL-AX was observed through a scanning electron microscope (FEI Nova, Nano SEM 450) equipped with an Everhart-Thornley Detector (ETD, low energy using secondary electrons) operated at 10 kV. For SEM analysis, CL-AX was suspended in deionized water and then lyophilized. Cross-sections of the lyophilized sample were then obtained through a sharp stainless steel blade and placed on an aluminum stub mounted with silver paint. The sample was analyzed using SEM after gold sputtering with a sputter coater (Denton, desk V HP).

### 4.5. Dynamic and Equilibrium Swelling

For measuring the pH-dependent swelling, each formulation of CL-AX1, CL-AX2, and CL-AX3 was placed in separate cellophane bags and suspended in HCl buffer (pH 1.2), phosphate buffers (pH 6.8 and 7.4), and deionized water for 6 h [12]. After regular intervals, these bags were removed from their respective media and weighed after blotting the excess media. After that, the bags were placed again in the media to continue the swelling studies. The swelling capacity (g.g^−1^) was calculated using Equation (1). This experiment was conducted six times, and the mean value was reported.

(1) $$Swelling\;capacity\;\left(g/g\right)=\frac{W_{t}-W_{0}-W_{c}}{W_{0}}$$

where *W_t_*, *W_0_*, and *W_c_* were the weights of the swollen CL-AX along with the wet cellophane bag, the weight of the dry CL-AX, and the weight of the wet cellophane bag, respectively. 

The normalized degree of swelling (*Q_t_*) is the ratio of the swelling media penetrated into CL-AX to the initial weight of the CL-AX at time *t*, calculated using Equation (2).

(2) $$Q_{t}=\frac{W_{s}-W_{d}}{W_{d}}=\frac{W_{t}}{W_{d}}$$

where *W_s_* was the weight of the swollen CL-AX at time *t*, *W_d_* was the weight of the dried CL-AX at time t = 0, and *W_t_* was the weight of water penetrated into the CL-AX at time *t*. 

The normalized equilibrium degree of swelling (*Q_e_*) is the ratio of the swelling media penetrated into the CL-AX at *t_∞_* to the weight of the dried CL-AX at *t* = 0 and can be determined using Equation (3).

(3) $$Q_{e}=\frac{W_{\infty }-W_{d}}{W_{d}}=\frac{W_{e}}{W_{d}}$$

where *W*_∞_ was the weight of swollen CL-AX at time *t*_∞_ when the swelling remained constant, *W_d_* was the weight of the dried CL-AX at *t* = ∞, and *W_e_* was the amount of water absorbed by the CL-AX at *t*∞.

### 4.6. Swelling Kinetics

To find the kinetic order of swelling, the values of the normalized degree of swelling (*Q_t_*) and the normalized equilibrium degree of the swelling (*Q_e_*) were used [27]. For second order kinetics, Equation (4) was used.

(4) $$\frac{t}{Q_{t}}=\frac{1}{KQ_{e^{2}}}+\frac{t}{Q_{e}}$$

The plot between the *t/Q_t_* on the y-axis and the *t* on the x-axis should be a linear line with a slope of 1/*Q_e_* and an intercept 1/*kQ_e_*^2^. 

### 4.7. Swelling and Deswelling in Response to External Stimuli

The presence of hydrophilic groups (-OH,-COOH, CONH_2_, etc.) in polymers impart the swelling and deswelling properties against various stimuli, e.g., pH, salt, solvents, temperature, etc. [28,29]. To observe the phenomenon of swelling and deswelling (on–off switching) in CL-AX, a gravimetric method was applied [30]. A weighed quantity (0.1 g) from each of the three formulations was enclosed in a cellophane bag and immersed in deionized water for 1 h for swelling; the swelling capacity was determined using Equation (1) after regular intervals (every 15 min). Then, the same bag having swollen the CL-AX formulation was allowed to deswell in pure ethanol for 1 h and the decrease in swelling was calculated after 15 min using Equation (1). 

A similar experiment was performed to study the swelling–deswelling behavior of CL-AX in buffer solutions of pH 7.4 and 1.2. All three formulations of CL-AX were allowed to swell first in a buffer solution of pH 7.4 for 1 h and then suspended in a buffer solution of pH 1.2 to measure the deswelling capacity for 1 h. 

Similarly, CL-AX formulations were again allowed to swell and deswell in water and an aqueous solution of NaCl (0.9% *w*/*v*, normal saline), respectively, and the swelling and deswelling capacity was determined using Equation (1). These swelling–deswelling (on–off) experiments of CL-AX in different media, i.e., water/ethanol, buffer solutions of 7.4/1.2, and water/normal saline were repeated three times, and the mean values were recorded.

### 4.8. Biocompatibility

The compatibility of a material with living tissues is essential to establish the pharmaceutical and biomedical applications of a new material. For the appraisal of the biocompatibility of CL-AX, hemocompatibility (hemolytic potential and thrombogenicity) was determined using the directions and protocol described by International Standard Organization (ISO) (ISO10993-4, 1999).

#### 4.8.1. Hemolytic Potential of CL-AX2

Hemolytic potential of CL-AX2 was determined through the direct interaction of the material with the blood. The procedure adopted for hemolysis was explained by the American Society for Testing and Materials [31,32]. Briefly, the sample (CL-AX2 powder, 500 mg) was allowed to mix with phosphate buffer saline (PBS) and then incubated at 37 °C for 24 h. After washing with PBS, the CL-AX2 was incubated again for 3 h at 37 °C with a known concentration of citrate blood and PBS. At the end of the incubation period, the mixture was processed through centrifugation at 10,000 rpm for 15 min. The supernatant was separated and scanned through a UV-Vis spectrophotometer at 540 nm to determine the optical density (OD) using Equation (5). For the positive and negative control, a known concentration of citrate blood was incubated with distilled water and with PBS, respectively.

(5) $$Hemolytic\;index\;\left(\%\right)=\frac{OD\;of\;test\;sample-OD\;of\;negative\;control}{OD\;of\;positive\;control-OD\;of\;negative\;control}\times 100$$

#### 4.8.2. Thrombogenicity Evaluation

The thrombogenicity potential (thrombus formation) of CL-AX2 was determined using an already reported method [32,33]. CL-AX2 (500 mg) was mixed with PBS and then incubated for 24 h at 37 °C. After decanting the excess PBS from the CL-AX2 mixture, citrate blood (2 mL) and CaCl_2_ solution (0.1 M, 0.2 mL) were added and allowed to clot for 45 min. Distilled water was added to stop the clotting process, and the formed clots were removed. These clots were fixed with formaldehyde (36–38%, 5 mL), dried, and weighed. The concentration of thrombose was calculated using equation (6). For the positive and negative control, the same process was repeated without the addition of CL-AX2, and without citrate blood and CL-AX2, respectively.

(6) $$Thrombose\;\left(\%\right)=\frac{mass\;of\;test\;sample-mass\;of\;negative\;control}{mass\;of\;positive\;control-mass\;of\;negative\;control}\times 100$$

### 4.9. In Vitro Drug Release Study

#### 4.9.1. Preparation of Sustained Release Tablets 

CL-AX2 was used as a matrix system to develop the sustained release tablets of enalapril maleate. Enalapril maleate belongs to the biopharmaceutical classification system (BCS) class III, i.e., high solubility and low permeability, which is used to control high blood pressure. For better control of high blood pressure, a constant therapeutic level should be maintained in the blood. Therefore, a sustained release dosage form with zero order release kinetics would be an ideal drug delivery system of enalapril maleate to control high blood pressure by maintaining a constant concentration in the blood. For the sustained release formulation of enalapril maleate, CL-AX2 (100 mg) was mixed with the drug (20 mg) and a kneading process was carried out through aqueous PVP K30 (5% *w*/*v*). The prepared damp mass was dried at 60 °C and passed through sieve no. 20 to obtain uniformed size granules. The dried granules were lubricated with magnesium stearate (5 mg) and then compressed to a 7 mm diameter tablet using flat surface punches. During compression, the hardness of the tablets was maintained at 6–8 kg/cm^2^. 

#### 4.9.2. Drug Release Study 

The in vitro drug release study from the CL-AX2 tablets was carried out at pH 6.8 using a USP Dissolution Apparatus II operating at 50 rpm and 37 ± 0.5 °C. Aliquots of the sample (5 mL) were withdrawn after regular intervals and immediately replenished with freshly prepared buffer of pH 6.8. The sample was filtered, diluted (if required), and scanned through a UV-Visible spectrophotometer at 216 nm.

#### 4.9.3. Drug Release Kinetics and Mechanism 

The drug release kinetics from the CL-AX2 matrix tablets was evaluated through the zero-order model (Equation (7)) and the release mechanism through the Korsmeyer-Peppas model (Equation (8)) [25,26,34]. The highest value (≈1) of the regression coefficient (R^2^) was considered the best fit model.

(7) $$Q_{t}=K_{0}t$$

where *K*_0_ indicated the zero order rate constant, and *Q_t_* was the amount of drug released in time *t*.

(8) $$\frac{M_{t}}{M_{\infty }}=K_{p}t^{n}$$

where *M_t_/M*_∞_ explained the amount of drug released in time *t*, *K_p_* was the power-law constant, and *n* denoted the release exponent. The value of *n* was used to establish the underlying mechanism of drug release. The drug release mechanism followed the Fickian diffusion, non-Fickian diffusion, case-II transport, and super case-II transport if the value of *n* was <0.45, >0.45 and <0.89, equal to 0.89, and >0.89, respectively [25,26].

## Figures and Tables

**Table 1:** Crosslinking of AX via Ca^2+^ ions by reacting with CaCl_2_.

Sample	AX (g)	CaCl_2_ (g)	Yield (%)	Solubility	Nature in DW, pH 1.2, pH 7.4
CL-AX1	0.25	0.5	68	DMSO, DMAc	Transparent thick gel
CL-AX2	0.25	1.0	76	DMSO, DMAc	Transparent thick gel
CL-AX3	0.25	1.5	78	DMSO, DMAc	Transparent thick gel

**Table 2:** Results of hemocompatibility studies of CL-AX2.

Parameters	Observations		Conclusion
Thrombus formation	Weight of blood clot (g) 0.39 ± 0.03	Thrombose (%) 93.79 ± 1.97	Non-thrombogenic
Hemolytic potential	OD ^1^ 0.22 ± 0.04	Hemolytic index (%) 2.71 ± 0.44	Nonhemolytic

^1^ Optical density.

**Table 3:** Mathematical data of drug release kinetics.

Formulation Code	Zero Order		Korsmeyer-Peppas Model		
	K_0_	R^2^	K_KP_	*n*	R^2^
CL-AX2	4.078	0.9911	5.107	0.923	0.9931
